# Genotype-phenotype correlation in patients with 21-hydroxylase deficiency

**DOI:** 10.3389/fendo.2023.1095719

**Published:** 2023-03-13

**Authors:** Peng Tang, Jun Zhang, Song Peng, Yapeng Wang, Haoyang Li, Ze Wang, Yao Zhang, Yiqiang Huang, Jing Xu, Dianzheng Zhang, Qiuli Liu, Luofu Wang, Weihua Lan, Jun Jiang

**Affiliations:** ^1^ Department of Urology, Daping Hospital, Army Medical University, Chongqing, China; ^2^ Fifteen Squadron Five Brigade, School of Basic Medical Science, Army Medical University, Chongqing, China; ^3^ Department of Bio-Medical Sciences, Philadelphia College of Osteopathic Medicine, Philadelphia, PA, United States

**Keywords:** congenital adrenal hyperplasia, 21OHD, genotype, phenotype, CYP21A2

## Abstract

**Introduction:**

21-hydroxylase deficiency (21OHD) is the most common cause of congenital adrenal hyperplasia (CAH). However, patients with 21OHD manifest various phenotypes due to a wide-spectrum residual enzyme activity of different CYP21A2 mutations.

**Methods:**

A total of 15 individuals from three unrelated families were included in this study. Target Capture-Based Deep Sequencing and Restriction Fragment Length Polymorphism was conducted on peripheral blood DNA of the three probands to identify potential mutations/deletions in CYP21A2; Sanger sequencing was conducted with the DNA from the family members of the probands.

**Results:**

Dramatically different phenotypes were seen in the three probands of CAH with different compound heterozygous mutations in CYP21A2. Proband 1 manifested simple virilizing with mutations of 30-kb deletion/c.[188A>T;518T>A], the latter is a novel double mutants classified as SV associated mutation. Although both probands carry the same compound mutations [293-13C>G]:[518T>A], gonadal dysfunction and giant bilateral adrenal myelolipoma were diagnosed for proband 2 and proband 3, respectively.

**Conclusion:**

Both gender and mutations contribute to the phenotypes, and patients with the same compound mutations and gender could present with different phenotypes. Genetic analysis could help the etiologic diagnosis, especially for atypical 21OHD patients.

## 1 Introduction

Most congenital adrenal hyperplasia (CAH) resulted from deficiencies of the enzymes, encoded by different cytochrome P450 (CYP) genes, in adrenal steroidogenesis (1). In humans, six CYPs including *CYP11A1*, *CYP11B1*, *CYP11B2*, *CYP17A1*, *CYP19A1*, and *CYP21A2* are involved in the synthesis of steroid hormones and deficiencies in any of them can cause CAH. Of note, ~95% of CAH patients have a deficient 21-hydroxylase (21OH) due to the mutation of *CYP21A2*. Since adrenocorticotropic hormone (ACTH) is partially downregulated by glucocorticoid and mineralocorticoid through a feed-back mechanism, reduced levels of glucocorticoid and mineralocorticoid are accompanied by elevated level of ACTH, which in turn results in not only accumulation of precursors in adrenal androgenesis but also exacerbates CAH (2). However, a wide spectrum of clinical manifestations was observed in patients with different mutations of their CYPs.

Patients with 21OHD can be categorized into three subgroups based on their clinical phenotypes: classic salt‐wasting (SW), classic simple virilizing (SV), and the non-classic (NC) forms (3). SW is most severe and could be life‐threatening if occurring in early infancy. Classic SV is characterized by prenatal virilization in females and virilization in both sexes without salt wasting (4). NC usually shows mild symptoms of androgen excess and sometimes is even asymptomatic (5). The diverse phenotypes of 21OH-caused CAH are the results of varying degrees of enzymatic deficiency. For instance, Wilson RC et al. found that patients with mutations causing complete loss of enzymatic activity presented with more severe phenotype, whereas patients with partial loss of enzymatic activity exhibited non-classical CAH (6). Genotype-phenotype correlations in CAH have been reported previously (7, 8). In this study, we found that some phenotype is associated certain *CYP21A2* mutation, and sometimes the same compound heterozygous mutation of *CYP21A2*, can lead to different phenotypes. These specific cases add to our knowledge of genotype-phenotype correlations of *CYP21A2*.

## 2 Materials and methods

### 2.1 Patients

This study design was reviewed and approved by the ethics committee in the Daping Hospital of Army Medical University. A total of 13 individuals from three unrelated families with their medical records including computed tomography (CT) or magnetic resonance imaging (MRI) scan, and laboratory examinations as well as their family history were included in this study. All procedures were carried out in accordance with the ethical standards of the institutional research committee and with the 1964 Helsinki Declaration and its later amendments or comparable ethical standards. Written informed consent for the use of medical records and related images was obtained from each patient.

### 2.2 PCR-based restriction fragment length polymorphism

Total DNA isolated from the peripheral blood leukocytes were obtained from the 15 individuals and used for sequencing to identify potential mutations and large deletions in CYP21A2 gene. To identify large gene deletions, primers CYP779f and Tena32F (available upon request) were used to amplify a fragment of 8515-bp followed the manufacturer’s recommendations. The 8.5-kb PCR product was digested with TaqI restriction endonuclease and the digested products were analyzed by electrophoresis in agarose gels.

### 2.3 DNA sequencing and analyses

Target Capture-Based Deep Sequencing (AmCare Genomic Laboratory, Guangzhou, Guangdong, China) was conducted with the PCR product amplified by CYP779f and Tena32F. The sequencing was carried out on NGS platform (HiSeq X system; Illumina) with PEx150 read length according to the manufactory’s instructions. Bioinformatics analyses were performed with in-house pipeline to identify rare or novel variants of CYP21A2 with Gnomad, HMGD, ClinVar, dbSNP for filtering and computational prediction algorithms. Software PolyPhen-2, SIFT, and MutationTaster were used for pathogenesis evaluation (**Supplementary Table 1**).

After the identification of the variants in the probands, Sanger sequencing were conducted to determine family co-segregation. The corresponding primers were designed by Primer 3.0 and available upon request. The final pathogenicity of the variants was estimated using the American College of Medical Genetics and Genomics guidelines.

## 3 Results

### 3.1 The probands

Proband 1 was an 8-year-old girl who was diagnosed as congenital adrenal hyperplasia when she was born with an atypical genitalia but no salt-wasting symptom in another hospital. She underwent clitoroplasty in that hospital at the age of 8 months and glucocorticoid and mineralocorticoid replacement therapy was prescribed. Laboratory investigations revealed elevated levels 17-hydroxyprogesterone (17-OHP, >75.75 nmol/L, normal 0.2–3.0 nmol/L) androgens, testosterone, and ACTH as shown in **Table 1**. Her karyotype is 46, XX.

**Table 1:** Laboratory parameters of the probands.

	Reference range	Proband 1 (female)		Proband 2 (male)		Proband 3 (male)
		After birth	During readmission	On admission	After hormone supplement	
17-OHP (nmol/L)	0.2–3.0	>75.75	–	485.20	–	–
DHEA (ng/mL)	1.70-6.10	–	–	7.99	–	–
Testosterone (ng/mL)	male:1.75-7.81 female: 0-0.75	3.41	7.60	4.05	1.86	–
DHT (pg/mL)	250.0-990.0	–	–	1777.99	–	–
Androstenedione (ng/mL)	0.25–1.21	>10	–	–	–	–
ACTH (pg/mL)	4.7-48.8	–	1131.00	136.0	–	–
Cortisol (nmol/L)	185-624	–	199.6	212	–	–
Aldosterone (ng/dL)	Supine:3.0–23.6/ Standing:3.0–35.3	–	3.45/2.95	14.9/11.4	–	–
FSH (mIU/mL)	1-8	3.17	0.23	0.25	6.40	–
LH (mIU/mL)	1.24-8.62	0.25	<0.20	<0.20	3.18	–
Na^+^ (mmol/L)	137.0-147.0	–	140.9	140.3	–	141.2
K^+^ (mmol/L)	3.5-5.3	–	4.08	3.98	–	4.52
17-OH (umol/24H)	8.3-33.2	–	–	–	–	38.5
17-KS (umol/24H)	20.8-76.3	–	–	–	–	98.8

17-OHP, 17-hydroxyprogesterone; DHEA, dehydroepiandrosterone; DHT, dihydrotestosterone; ACTH, adrenocorticotropic hormone; FSH, follicle-stimulating hormone; LH, luteinizing hormone; 17-OH, 17-hydroxycorticosteroid; 17-KS, 17-ketosteroid.

**Figure 1:**
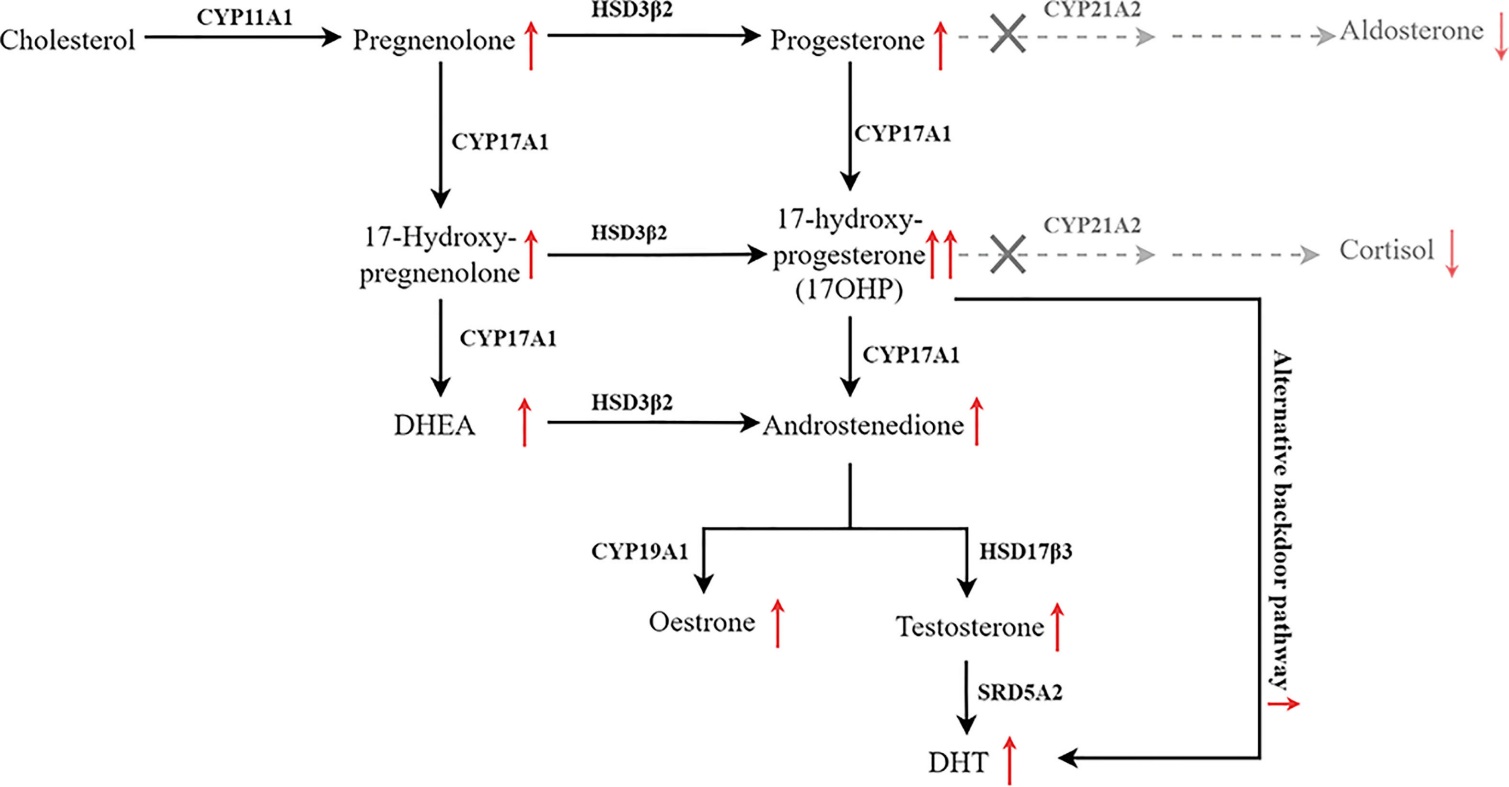
Pathway of steroid biosynthesis in the adrenal cortex. 21-hydroxylase deficiency results in decreased cortisol and aldosterone, and elevated cortisol precursors and adrenal androgens. The grey denotes deficient pathways.

She was referred to our hospital due to virilization and presented with high stature (140 cm tall, >97th percentile of girls of Han nationality in the same age group in China) and deep voice. Physical examination identified clitoral enlargement, partially fused labia majora, and a urogenital sinus in place of separate urethral and vaginal openings. She was taking hydrocortisone (20 mg per two days) before admission to our hospital. We noticed that based on her weight of 41 kg, she should be treated with 12.7-19.1 mg in a day according to the guideline (9). Laboratory examination revealed a markedly elevated testosterone level of 7.60 ng/mL and CT identified hyperplasia of bilateral adrenal gland, and the cavernous structure in the perineum as shown in **Figure 1**. She was advised to maintain her regular replacement therapy for now and will have genitoplastic surgery and vaginoplasty when she becomes an adult. None of her parents has any related symptom.

**Figure 2:**
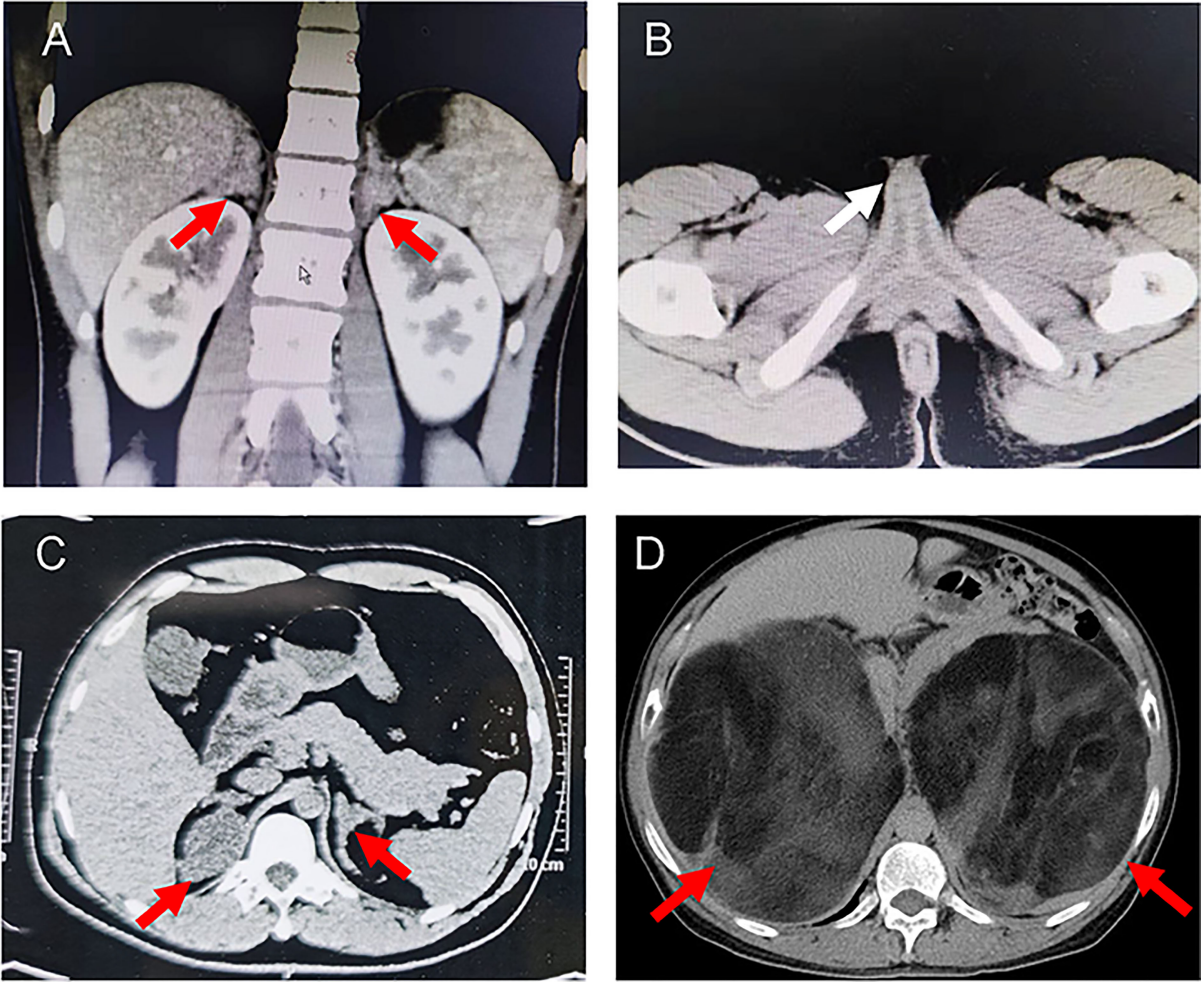
Representative computed tomography (CT) scans of the probands. **(A)** CT images showing bilateral adrenal hyperplasia in the proband 1. **(B)** Pelvic CT image of proband 1 indicated the cavernous structure in the perineum (white arrow). **(C)** CT images showing left adrenal hyperplasia and right adrenal adenomatous hyperplasia (4.3*3.7 cm) in the proband 2. **(D)** CT image showing giant bilateral adrenal masses (left: 20*25 cm; right: 30*40 cm) in the proband 3. The red arrows indicate the adrenal lesions.

Proband 2 was a 37-year-old male referred to our hospital due to the azoospermia, without other symptoms. He had been diagnosed as idiopathic hypogonadotropic hypogonadism (IHH) based on small testes and significantly decreased serum levels of FSH (0.07 mIU/mL) and LH (0.00 mIU/mL, **Table 1**) but without salt wasting. The patient had sex premature with masculinization and precocious pubarche when he was only 7 years old. He had a short stature (149 cm in height), small testis (left: 11 mL; right: 12 mL), and mild hypospadias. CT scan revealed left adrenal hyperplasia and right adrenal adenomatous hyperplasia (4.3*3.7 cm, **Figure 2**). Scrotal ultrasound detected an 8 mm*6mm mass in his left testis, likely to be testicular adrenal rest tumors (TARTs). Laboratory tests showed elevated serum 17OHP (485.20 nmol/L, normal 0.2–3.0 nmol/L), dehydroepiandrosterone (DHEA) (7.99ng/mL, normal 1.70-6.10), and dihydrotestosterone (DHT) (1777.99 pg/mL, normal 250.0-990.0, **Table 1**). Gonadotropin-releasing hormone (GnRH) stimulation test was negative (**Supplementary Table 2**). We concluded that he has congenital adrenal hyperplasia and secondary hypogonadotropic hypogonadism. After eight months of replacement therapy, his semen count was relatively normal (**Supplementary Table 3**). His parents were normal.

Proband 3 was a 59-year-old male admitted to our hospital with marked abdominal distension. CT scan revealed giant bilateral myelolipoma (left: 20*25 cm; right: 30*40 cm; **Figure 2**). His 24-hour urine 17-hydroxycorticosteroid (17-OH) and 17-ketosteroid (17-KS) were a bit higher (**Table 1**). The proband underwent resection of his myelolipoma accompanied with glucocorticoid and mineralocorticoid replacement therapy. Patient history indicates that he had a short stature at birth but no salt wasting. Both his parents died with unknown causes. His brother also underwent resection of bilateral giant myelolipomas at age of 50. But his three sisters and three daughters appear to be normal.

### 3.2 Genetic mutations

**Figure 3:**
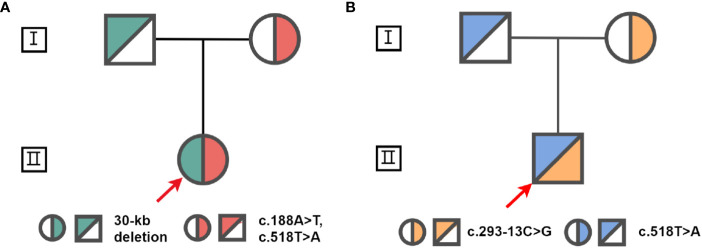
Pedigrees for families of proband 1 **(A)** and s proband 2 **(B)**. The red arrow indicates the probands.

Proband 1 inherited a 30kb deletion (a classic category of chimeric *CYP21A1P*/*CYP21A2* genes: CH-2, which carries common mutations including: c.92C>T, p.P31L; c.188A>T, p.H63L; c.293-13C>G; c.332_339del, p.G111Vfs*21; c.518T>A, p.I173N) (10) and a double mutants *in cis* (c.[188A>T;518T>A], p.[H63L;(I173N)]) (**Figure 3**). Both proband 2 and 3 have a compound heterozygous mutation of *CYP21A2* (c.293-13C>G and c.518T>A, p.I173N, **Figure 3**), the detailed pedigree for family of proband 3 has been previously reported (11). We found that proband 2 inherited c.293-13C>G from his mother and c.518T>A, p.I173N from his father. However, we cannot determine his genetic heritage since both his parents are dead. His brother harbored the same compound mutations. Each of the other family members of the three probands harbored a monoallelic *CYP21A2* variant. The genotypes and phenotypes of the individuals were summarized in **Table 2** and **Supplementary Table 4**.

**Table 2:** The genotype-phenotype of the probands.

Patients	1	2	3
Genotype	c.[188A>T;518T>A], 30-kb deletion	c.293-13C>G, c.518T>A	c.293-13C>G, c.518T>A
Sex	Female	Male	Male
Age (year)	8	37	59
Virilization	+	–	–
Dehydration	–	–	–
Short stature	–	+	+
Premature growth of pubic hair	–	+	–
Fertility	N/A	–	+
Appearance of genital	Clitoral hypertrophy	Small testes	Almost normal
TART or OART	–	+	–
Adrenal hyperplasia	Bilateral hyperplasia	Bilateral hyperplasia	Bilateral myelolipomas
17-OHP (nmol/L)	>75.75	485.20	N/A

17-OHP, 17-hydroxyprogesterone; TART, testicular adrenal rest tumor; OART, ovarian adrenal rest tumor; N/A, not applicable. +, with the symptom; -, without the symptom.

## 4 Discussion

CAH is an autosomal recessive disease caused by deficient enzymes including 21OH, 11β-hydroxylase, 17α-hydroxylase, 3β-hydroxysteroid dehydrogenase type 2, steroidogenic acute regulatory protein, and P450 oxidoreductase (12). 21OHD-caused CAH is found 1 in every 10 000 to 20 000 new born (13) with diverse phenotype. Complete inactive *CYP21A2* results in deficiencies of both glucocorticoid and mineralocorticoid, and severe adrenal-derived androgen excess. In newborn females, excess androgen is clinically evident due to virilization of their external genitalia. However, males with excess androgen appear to be normal at birth but with premature growth of pubic and axillary hair, oily skin, rapid somatic and skeletal maturation. Proband 1 described in this study is a girl and diagnosed after birth, who carried compound mutations with the 30-kb deletion inherited from her father and c.[188A>T;518T>A], a novel double mutants *in cis* (14), from her mother. The c.188A>T mutation has a mild effect on the enzyme; whilst the c.518T>A affects the enzymatic activity more dramatically (15, 16). The double mutants in the young girl (proband 1) appears to be associated with simple virilization according to her clinical presentations. On the other hand, both probands 2 and 3 in this study are male and their azoospermia and giant bilateral adrenal myelolipomas were not recognized until much later. Thus, the gender could be one of the main factor that contributes to the diverse phenotype (1, 17).

Findings from previous studies indicate that phenotypes of 21OHD-caused CAH maybe mutation site-specific. When the genotype-phenotype correlation was examined with more than 230 mutations (18), 70–75% of the CAH cases were caused by the ten most common variants (7, 19, 20). The p.V282L, one of the ten most common variants, accounts for 0.9% cases in Chinese people, compared with 26.2% and 23.9% in Argentinian and other heterogeneous western population respectively (21). In this study, we found that proband 2 and 3 have the same compound mutations [293-13C>G]:[518T>A] but totally different clinical manifestations. Previous study reported patients with this compound heterozygous mutation shows phenotypic variability (NC : SV:SW = 1:36:13) (7). In our report, proband 2 presented with azoospermia mainly due to secondary gonadal dysfunction. The proband has been initially misdiagnosed as IHH, a rare type of congenital disease characterized with dysfunction in the secretion of hypothalamic GnRH and reduced serum levels of sex steroids (22). In fact, the lower normal range of testosterone with suppressed gonadotropins is also the typical profile of secondary hypogonadotropic hypogonadism (23). Previous studies have reported that the accumulation of excessive progesterone, 17OHP, estrogen, and androgens jointly contribute to hypothalamic-pituitary -gonadal (HPG) axis suppression and the secondary gonadal failure (23–25). Based on the elevated levels of 17OHP, DHT, and DHEA, proband 2 is diagnosed as CAH and nearly full recover of semen count after glucocorticoid supplementary therapy further confirmed his gonadal dysfunction is secondary to hypogonadotropic hypogonadism. In addition, the testicular mass might be TART, since it is well known that upregulated ACTH secretion causes TARTs (25). Previous study has shown that hypogonadism and azoospermia could be associated with bilateral TARTs (26). Noteworthy, proband 2 had relatively high levels of DHT, which might be due to the activation of alternative backdoor pathway as described previously (27). Although carrying the same compound mutations, proband 3 presented with giant bilateral adrenal myelolipomas, which are caused by chronic ACTH hyperstimulation (28). One of the potential explanations is that different c.293-13C>G were transcribed in these patients (29).

Glucocorticoid replacement is a life-long treatment and the options for glucocorticoid are hydrocortisone or long-acting synthetic glucocorticoid (2). To avoid suppressive effect of growth and chronic cushingoid complication, hydrocortisone is usually prescribed for children (30). The long-acting synthetic glucocorticoid is generally recommended for adolescents and adults (31). Although hydrocortisone was prescribed for proband 1, virilization has developed progressively. This could be the result of insufficient dosage and/or unresponsive surveillance (9). Intensified glucocorticoid treatment is the first choice for gonadal dysfunction (24). For proband 2, normal level of FSH after one month dexamethasone supplement suggests the rehabilitation of HPG axis. After eight months of treatment his sperm production became relatively normal. In addition, differences of sex development (DSD) often occur in females with classic 21OHD. Decision for sex mainly depends on karyotype and the degree of virilization. Timely diagnosis is the key for early treatment of CAH and the ratio between the intermediates and the hormones is the mainstay for the diagnosis. Since elevated 17OHP is a typical indicator of 21OHD-associated CAH (32), the levels of 17OHP should be monitored closely for individuals showing evidence of androgen excess.

In conclusion, we described three probands with diverse phenotypes of CAH carrying different compound heterozygous mutations of *CYP21A2*. The novel double mutants (c.[188A>T;518T>A]) is a SV subtype. In addition to be consistent with that genders and different mutations contribute to various phenotypes, we found that diverse phenotypes could be presented in patients with identical compound mutations in the same gender. Moreover, genetic analysis could help the etiologic diagnosis, especially for atypical 21OHD patients. These specific cases add to our knowledge of genotype-phenotype correlations of *CYP21A2.*

## Data availability statement

The original contributions presented in the study are included in the article/**Supplementary material**, further inquiries can be directed to the corresponding author/s.

## Ethics statement

The studies involving human participants were reviewed and approved by the ethics committee in the Daping Hospital. The patients/participants provided their written informed consent to participate in this study.

## Author contributions

Study design were conducted by JJ, WL, and JZ. Samples and clinical data were collected by YH, LW, YZ, JX, and YW. Analysis of data were performed by PT, SP, ZW, YZ, and HL. DZ, QL, JZ and PT wrote the manuscript which was revised and approved by all authors.
