# Supplementary material for: Genotype-phenotype correlation in patients with 21-hydroxylase deficiency
Source: Front Endocrinol (Lausanne). 2023 Mar 13;14:1095719. doi: 10.3389/fendo.2023.1095719 (PMC10042299; doi:10.3389/fendo.2023.1095719)
Supplement: Supplementary file 1 [file DataSheet_1.docx]

Supplementary Material

# Supplementary Tables

Supplementary table 1. The results of PolyPhen-2/SIFT/MutationTaster/ACMG analysis about the mentioned mutations.

| Changes in nucleotide | Changes in the amino-acid | PolyPhen-2 | SIFT | MutationTaster | ACMG |
| --- | --- | --- | --- | --- | --- |
| c.92C>T | p.P31L | Benign | Tolerated | Disease causing | likely pathogenic |
| c.188A>T | p.H63L | Benign | Tolerated | Polymorphism | likely pathogenic |
| c.293-13C>G | - | - | - | - | pathogenic |
| c.332_339del | p.G111Vfs*21 | - | - | - | pathogenic |
| c.518T>A | p.I173N | Probably damaging | Deleterious | Disease causing | likely pathogenic |
| 30-kb deletion | - | - | - | - | pathogenic |

Supplementary table 2. Basal and stimulated (GnRH) FSH and LH levels of proband 2.

|  | FSH (mIU/mL) | LH (mIU/mL) |
| --- | --- | --- |
| Basal | 0.22 | <0.20 |
| 30 min | 1.49 | 1.01 |
| 60 min | 1.59 | 0.76 |
| 90 min | 1.75 | 0.63 |

Supplementary table 3. Semen analysis of proband 2.

| Parameter | Reference range | Date | |
| --- | --- | --- | --- |
|  |  | 23-02-2021 | 31-07-2022 |
| Semen volume (mL) | 1.5-6.0 | 2.0 | 3.0 |
| Semen pH | 7.2-8.0 | 7.2 | 7.2 |
| Sperm concentration (×10^6/ml) | ≥15 | 0 | 27.9 |
| PR (%) | ≥32％ | 0 | 37.2 |
| PR+NP (%) | ≥40％ | 0 | 3.3 |
| Normal morphological sperm (%) | ≥4％ | 0 | 48.1 |

PR, progressive motility; NP, non-progressive motility.

Supplementary table 4. The genotype-phenotype of the mutation carriers in three families.

| Families | 1 | |  | 2 | |  | 3 | | | | | | | | | |
| --- | --- | --- | --- | --- | --- | --- | --- | --- | --- | --- | --- | --- | --- | --- | --- | --- |
| Family members | Father | Mother |  | Father | Mother |  | Father | Mother | Brother | Sister | Daughter 1 | Daughter 2 | Daughter 3 | Niece | Granddaughter 1 | Granddaughter 2 |
| Genotype | 30-kb deletion/W | c.[188A>T;  518T>A] /W |  | c.518T>A/W | c.293-13  C>G/W |  | N/A | N/A | c.293-13  C>G/c.518T>A | c.518T>A/W | c.293-13  C>G/W | c.518T>A/W | c.293-13  C>G/W | c.293-13  C>G/W | W/W | c.518T>A/W |
| Dehydration | - | - |  | - | - |  | - | - | - | - | - | - | - | - | - | - |
| Virilization | - | - |  | - | - |  | - | - | - | - | - | - | - | - | - | - |
| Short stature | - | - |  | - | - |  | - | - | - | - | - | - | - | - | - | - |
| Premature growth of pubic hair | - | - |  | - | - |  | - | - | - | - | - | - | - | - | - | - |
| Fertility | Normal | Normal |  | Normal | Normal |  | Normal | Normal | Normal | Normal | Normal | Normal | Normal | Normal | Normal | Normal |
| Appearance of genital | Normal | Normal |  | Normal | Normal |  | Normal | Normal | Normal | Normal | Normal | Normal | Normal | Normal | Normal | Normal |
| Adrenal hyperplasia | - | - |  | - | - |  | - | - | Bilateral adrenal myelolipomas | - | - | - | - | - | - | - |
| 17-OHP (nmol/L) | N/A | N/A |  | N/A | N/A |  | N/A | N/A | N/A | N/A | N/A | N/A | N/A | N/A | N/A | N/A |

W, wild-type allele; 17-OHP, 17-hydroxyprogesterone; N/A, not applicable.
